# YPL-SLAM: A Simultaneous Localization and Mapping Algorithm for Point–line Fusion in Dynamic Environments

**DOI:** 10.3390/s24144517

**Published:** 2024-07-12

**Authors:** Xinwu Du, Chenglin Zhang, Kaihang Gao, Jin Liu, Xiufang Yu, Shusong Wang

**Affiliations:** 1 College of Agricultural Equipment Engineering, Henan University of Science and Technology, Luoyang 471003, China; 220320261787@stu.haust.edu.cn (C.Z.); 210321041663@stu.haust.edu.cn (K.G.); 220320261813@stu.haust.edu.cn (J.L.); yxf840211@163.com (X.Y.); 2 Longmen Laboratory, Luoyang 471000, China; 13323939272@163.com; 3 Collaborative Innovation Center of Machinery Equipment Advanced Manufacturing of Henan Province, Luoyang 471003, China

**Keywords:** dynamic environment, visual SLAM, YOLOv5s, point–line fusion, YPL-SLAM

## Abstract

Simultaneous Localization and Mapping (SLAM) is one of the key technologies with which to address the autonomous navigation of mobile robots, utilizing environmental features to determine a robot’s position and create a map of its surroundings. Currently, visual SLAM algorithms typically yield precise and dependable outcomes in static environments, and many algorithms opt to filter out the feature points in dynamic regions. However, when there is an increase in the number of dynamic objects within the camera’s view, this approach might result in decreased accuracy or tracking failures. Therefore, this study proposes a solution called YPL-SLAM based on ORB-SLAM2. The solution adds a target recognition and region segmentation module to determine the dynamic region, potential dynamic region, and static region; determines the state of the potential dynamic region using the RANSAC method with polar geometric constraints; and removes the dynamic feature points. It then extracts the line features of the non-dynamic region and finally performs the point–line fusion optimization process using a weighted fusion strategy, considering the image dynamic score and the number of successful feature point–line matches, thus ensuring the system’s robustness and accuracy. A large number of experiments have been conducted using the publicly available TUM dataset to compare YPL-SLAM with globally leading SLAM algorithms. The results demonstrate that the new algorithm surpasses ORB-SLAM2 in terms of accuracy (with a maximum improvement of 96.1%) while also exhibiting a significantly enhanced operating speed compared to Dyna-SLAM.

## 1. Introduction

Visual Simultaneous Localization and Mapping (V-SLAM) plays a critical role in the autonomous navigation of mobile robots. With advancements in robotics, an increasing number of researchers are directing their focus towards SLAM technology [1,2,3]. Classical vision SLAM systems typically include sensor data acquisition, visual odometry, backend optimization, map construction, and loop closure detection within their frameworks [4]; these include PTAM [5], ORB-SLAM2 [6], ORB-SLAM 3 [7], VINS-Mono [8], VINS-Fusion [9], etc. These algorithms are relatively stable in static environments; however, in dynamic environments, the interference of dynamic objects can trigger map drift and complicate data association, which greatly affects the positioning accuracy and map creation of the system. The miscorrelation of the data may even cause the system to crash. Removing dynamic feature points in dynamic environments can reduce the number of feature points, which can lead to object loss [10]. In scenarios with missing texture or motion blur, line features show greater robustness and can represent the structural features of the scene, providing intuitive visual information [11]. Therefore, it is crucial to study the extraction of line features for point–line fusion in dynamic environments. Such research could help to address challenges such as missing map point tracking, degraded localization accuracy, and map drift in dynamic scenes.

To address the problems of dynamic environments, which may lead to system tracking failures and degraded localization accuracy, we propose a SLAM system called YPL-SLAM. It is designed to address system failures and degraded localization accuracy due to insufficient feature point extraction or image blurring in dynamic environments. The YPL-SLAM algorithm combines the YOLOv5s algorithm with the point–line feature fusion technique. This greatly improves the localization accuracy of the system in dynamic scenes, enabling it to extract line features in non-dynamic regions while removing dynamic feature points to ensure that sufficient features are extracted to increase the stability of the system. We improve the ORB-SLAM2 algorithm by detecting objects in the environment through target detection and region segmentation threads, identifying dynamic, potentially dynamic, and static regions in the image, and determining the state of the potentially dynamic situation. Meanwhile, we remove dynamic feature points in the dynamic region and then extract line features in the non-dynamic region. Line features can more comprehensively represent structural features [12]. The main contributions of our work are summarized as follows:

1. We design an improved algorithm, YPL-SLAM, based on ORB-SLAM2, which combines YOLOv5s with point–line feature fusion technology, increases the target detection and region delineation threads, and adopts multi-thread parallelism to ensure the real-time performance and accuracy of the algorithm in a dynamic environment.
2. We construct a method to determine the potential dynamic region. The dynamic region, potentially dynamic regions, and static regions are obtained after target detection and area division. Through processing, the states of the potential dynamic region are determined and the dynamic feature points of the dynamic region are eliminated.
3. We use the weighted fusion strategy considering the image dynamic score and the number of successful feature point and line matches for point–line fusion optimization and processing. The line feature points in the static region are extracted and the point and line feature weights are processed according to the image dynamic score and the number of successful feature point–line matches to obtain the optimized point–line fusion results.

## 2. Related Works

Currently, the visual SLAM algorithms used for visual odometry are divided into three main categories: the feature point method, the optical flow method, and the direct method. The feature point method extracts feature points between consecutive frames and matches them; finally, it calculates the camera motion through the matching relationship. The optical flow method extracts feature points without matching and solves the camera motion based on the assumption of grayscale invariance. The direct method calculates the camera motion by considering the pixel positions of the spatial points in two consecutive frames based on the optical flow method, as well as building an error model.

The most commonly used algorithms for the extraction of feature points are SIFT [13], SURF [14], and ORB [15]. While these methods boast high accuracy, they are susceptible to instability or even failure when deployed in complex environments. Unlike these feature point methods, the direct methods utilize pixel-level grayscale image information to estimate the motion and scene structure by minimizing photometric errors. These methods are usually more robust in weakly textured scenes. Examples include LSD-SLAM [16], SVO [17], and DSO [18]. However, the direct methods include the assumption of grayscale invariance, and the performance of these methods suffers in scenes with significant changes in illumination. As a direct method, SLAM relies on an image gradient search to optimize the pixel gray values. However, due to the highly non-convex nature of images, particularly when significant camera motion is involved, the algorithm becomes susceptible to local minima. To address these problems, line feature-based SLAM methods have been proposed. Rong et al. [19] introduced PEL-SLAM, a point–line SLAM method leveraging ED-line [20] to enhance environmental adaptation and accuracy. However, this approach simultaneously escalates the computational complexity and heightens the sensitivity to dynamic scenes and illumination variations. Gomez-Ojeda et al. [21] introduced PL-SLAM, a visual SLAM system that integrates both point and line features. This fusion of features enables PL-SLAM to exhibit improved adaptability across diverse environments, particularly in scenarios with limited features or structured scenes. Zhang et al. [22] introduced PL-GM, a SLAM algorithm that merges 3D points and lines generated by RGB-D cameras with 2D point and line features. PL-GM achieves comparable or better performance compared to SLAM methods based on point–line features and point features. However, these methods may exhibit instability or subpar performance when dealing with moving objects or when there are dynamic changes in the scene. Given the important advances in deep learning for target detection, SLAM, combined with deep learning techniques, could be more effective in dynamic environments.

In dynamic environments, the primary objective of the visual SLAM system is to effectively distinguish dynamic targets from outliers. This involves integrating SLAM techniques with deep learning to leverage the capabilities of deep learning models for accurately filtering out dynamic information. This section outlines the related research in this area. On the one hand, SLAM is combined with semantic segmentation techniques. For example, the Dyna-SLAM method, proposed by Bescos et al. [23], combines the Mask R-CNN [24] algorithm with ORB-SLAM2 to effectively detect and segment dynamic objects. However, the method relies on target detection and semantic segmentation for localization tracking, which poses challenges, especially in real-time applications. Wang et al. [25] combined the visual inertial SLAM technique with deep learning to determine the motion probabilities of feature points by using semantic information extracted from images. This improves localization accuracy by eliminating feature points with an overly high motion probability. However, this motion probability grading model is constructed based on life experience and may be limited in multi-target motion scenarios. Yu et al. [26] introduced DS-SLAM, a semantic system that mitigates the influence of dynamic objects through a combination of semantic segmentation and optical flow techniques. However, a decrease in accuracy occurs when there is dense sampling in large-format images. On the other hand, SLAM is combined with target detection techniques. For example, Wu et al. [27] proposed YOLO-SLAM, which combines target detection methods and geometric constraint methods in a tightly coupled manner, aiming to reduce the impact of dynamic targets by adding a lightweight YOLOv3 network into the SLAM system. However, the lightweight YOLOv3 network has limitations regarding dynamic target recognition in complex scenes. Zhang et al. [28] employed a lightweight YOLOv5s network to enhance the system’s operation speed. Additionally, they integrated a pyramid-shaped scene parsing network split header at the YOLOv5s network’s head to achieve semantic extraction within the environment. However, the fast detection speed of the lightweight YOLO target detection algorithm may lead to less accurate results for small targets or heavily occluded targets. Song et al. [29] introduced YF-SLAM, a method leveraging the YOLO-Fastest network to rapidly identify dynamic target regions within dynamic scenes. Subsequently, they employed depth geometric constraints to filter out dynamic feature points, thereby ensuring the real-time operation of the SLAM system. However, when the camera moves in a blurred scene, the depth information is affected, which impacts the localization and map construction quality of the SLAM system. Gong et al. [30] proposed an algorithm for AHY-SLAM using the optical flow method with filtering of the input keyframes in each frame. It extracts the feature points of the keyframes using adaptive thresholding, eliminating the dynamic points using YOLOv5. However, when there are multiple moving objects in the scene, a large number of feature points are eliminated, and its robustness can be limited. Thus, these techniques exhibit poor performance or failure in scenarios involving multiple moving targets or motion blur in the camera. To address these issues, we propose YPL-SLAM, a point–line feature-based SLAM system for dynamic environments.

YPL-SLAM integrates the YOLOv5s object detection technology with point–line feature fusion, removes feature points from dynamic regions, extracts line features from static regions, and employs a weighted fusion strategy based on the image dynamic score and the number of successful matches of feature points and lines. This fusion aims to more efficiently detect and track moving objects in dynamic scenes, thereby reducing the negative impact of multiple dynamic objects on localization. Through this innovative approach, we expect to significantly improve the robustness and accuracy of SLAM systems when dealing with complex scenes exhibiting dynamic characteristics.

## 3. Methods

In this section, we introduce YPL-SLAM from four perspectives. First, we outline the general framework of YPL-SLAM. The main element of the general framework is the improvement of ORB-SLAM2. We then introduce target detection and region segmentation using the YOLOv5s algorithm, which improves the real-time performance of the system by identifying the region in which the target is located and dividing it into dynamic, potential, and static regions, as compared to commonly used algorithms that incorporate semantic segmentation. Then, the method for determining the state of the potential dynamic region is presented. Finally, we use the LSD [31] algorithm with the LBD [32] descriptor to extract the line features and matches within the static region and optimize the processing of point–line fusion using a weighted fusion strategy that considers the image dynamic score and the number of successful feature point–line matches.

### 3.1. Overview of the YPL-SLAM System

**Figure 1:**
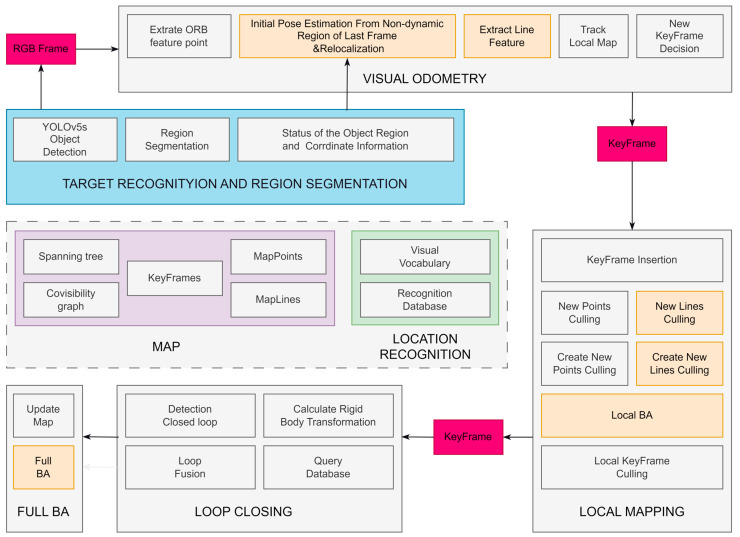
The SLAM framework of the YPL-SLAM algorithm, with the additional threads for target recognition and region delineation depicted in the blue box. Meanwhile, the yellow box illustrates the line feature extraction and processing module.

The ORB-SLAM2 algorithm consists of three threads: tracking, localization building, and loop closing. It utilizes ORB feature extraction for localization building, which combines FAST [33] key point detection and BRIEF [34] descriptors, offering rotational and scale invariance. While ORB-SLAM2 exhibits strong performance in static environments with localization accuracy, we chose to enhance it further. We add target detection and region delineation modules. At the same time, we add a module for the processing of the dynamic and potential dynamic regions in the tracking thread, determine the state of the potential dynamic region, remove dynamic feature points, extract non-dynamic region line features through these processes, and optimize the camera position via point–line fusion, as shown in Figure 1. The processing of the dynamic and potentially dynamic regions contributes to the better handling of moving objects during vision SLAM, and the point–line fusion approach used to optimize the camera position is expected to improve the robustness and localization accuracy of the system. Specific methods for these improvements will be detailed later.

### 3.2. Target Recognition and Region Segmentation

The YOLOv5s algorithm operates by taking an RGB-D image as input, extracting features through a convolutional neural network, and subsequently parsing these features to determine the target object’s category and bounding box coordinates. Unlike the two-stage detection algorithms of the RCNN series, YOLOv5s streamlines the process by eliminating the step of predicting the positions of candidate boxes. Instead, it treats the problem holistically as a regression task, utilizing convolutional networks for direct end-to-end model training. This approach significantly enhances the speed, surpassing that of other algorithms and ensuring the real-time operation of the entire SLAM system. By leveraging convolutional networks and direct end-to-end training, YOLOv5s accelerates target detection while also providing precise bounding box coordinate information. Moreover, it utilizes regression targets based on pixel information from the entire image, thereby minimizing false alarms in the background and enhancing the separation of the detected targets from background areas.

**Figure 2:**
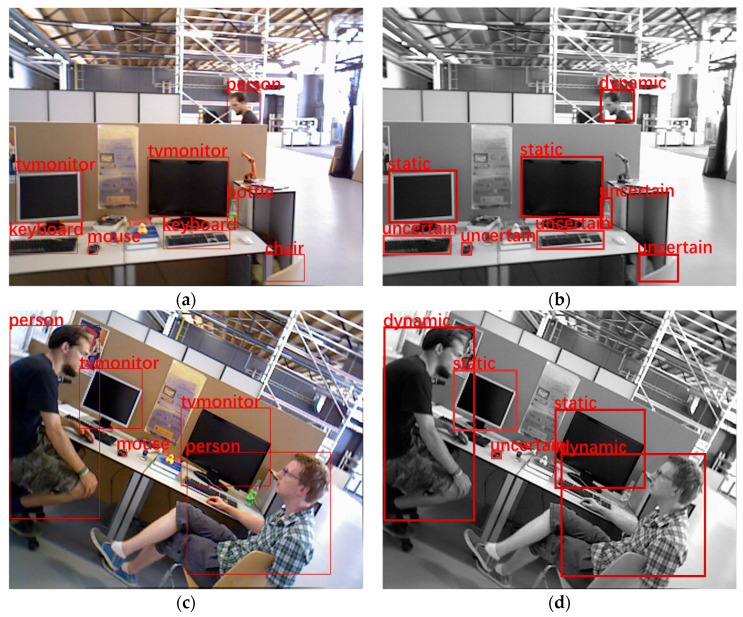
The YOLOv5s algorithm recognizes the targets, obtains the targets’ semantic information, and classifies these targets into static, dynamic, and potentially dynamic regions. (**a**,**b**) Recognizing the targets; (**c**,**d**) categorizing the area in which the target is located. Dynamic indicates a dynamic region, uncertain indicates a potential dynamic region, and static indicates a static region.

The YOLOv5s algorithm is used to detect the target in the image and obtain its bounding box location information, which is subsequently classified into three categories according to the region in which the target is located: static regions, potentially dynamic regions, and dynamic regions. The regions where the different targets are located are classified according to the classification criteria in Table 1. Targets with subjective mobility, such as humans and animals, are classified as dynamic regions, while objects that can easily be moved, such as mice, keyboards, and chairs, are classified as potentially dynamic regions, and objects that do not move easily, such as computer screens, cabinets, and windows, are categorized as static regions. Figure 2 illustrates the information about the coordinates of the regions obtained from the classification. Next, the segmented area’s coordinate information is sent to the tracking thread for subsequent target tracking and analysis. This process helps to identify and distinguish the different targets in the image more accurately and provides the basis for further dynamic region analysis. By categorizing the dynamic targets and potential dynamic regions, the system can respond more effectively to different target types, providing targeted information for subsequent processing. This integrated approach to target recognition and region segmentation is expected to improve the system’s ability to perceive and understand dynamic environments.

### 3.3. Determination of the State of a Potential Dynamic Region

**Figure 3:**
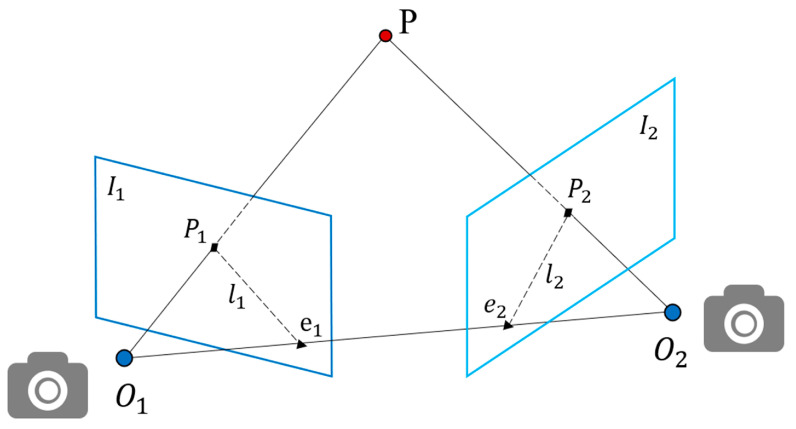
Geometric constraints on the poles.

According to the target detection and region delineation module, we can obtain the position information of the potential dynamic regions, dynamic regions, and static regions. The camera position is calculated using the extracted feature points, with the exception of the dynamic region, and RANSAC processing is performed to remove abnormal feature points. Then, the dynamic and potential dynamic regions are processed to obtain the state of the potential dynamic region, and the static feature points in the dynamic region are retained. The status of the region containing the object is determined through target detection. The base matrix F is computed for consecutive frames using ORB feature points extracted from areas outside the dynamic region. Geometric constraints are then applied to calculate the feature points within the potential dynamic regions, allowing for an assessment of the region’s status. The static feature points within the dynamic regions are retained. Figure 3 shows the geometric constraints on the poles.

We can obtain the Epipolar Constraint Formula as follows:

(1) E=t^RF=K−TEK−1x2TEx1=p2TFp1=0

In the equation, K represents the camera’s internal reference matrix, and R and t signify the camera’s motion from the previous frame coordinate system to the next frame coordinate system. $p_{1}$ and $p_{2}$ are characteristic points. $x_{1}$ and $x_{2}$ are normalized plane coordinates. F represents the fundamental matrix and E represents the essential matrix. We can obtain the following equation:

(2) $$l_{2}=\left(\begin{array}{l} a\\ b\\ c \end{array}\right)=Fp_{1}$$

We calculate the distance from the pixel point coordinate $p_{2}$ to the polar line $l_{2}$.

(3) $$d=\frac{\left|p_{2}^{T}l_{2}\right|}{\sqrt{a^{2}+b^{2}}}$$

where d denotes the distance from the point $p_{2}$ to the line $l_{2}$, where a, b, and c express the direction vectors of the line $l_{2}$.

Due to the effect of errors, we set a threshold for the distance from $p_{2}$ to the straight line $l_{2}$ and adjust the threshold according to the specific application scenario and needs. When the calculated d exceeds this threshold, the point is considered an anomaly; otherwise, the point is retained for subsequent calculations.

The polar geometric constraints allow us to determine the anomalous feature points, and the boundary information allows us to determine the number of feature points extracted from different areas. We remove the dynamic feature points for dynamic regions, while, for potentially dynamic regions, the state is then determined according to the following equation:

(4) $$T=\frac{1}{1+e^{-(N_{m}+1)/N_{a}}}$$

where $N_{m}$ denotes the number of abnormal points in the center of the potential dynamic region, and $N_{a}$ denotes the number of all feature points in the potential dynamic region. When the calculation result exceeds the set threshold ε, it is considered that the region is dynamic; otherwise, the target is considered stationary.

### 3.4. Extraction of Non-Dynamic Region Line Features

**Figure 4:**
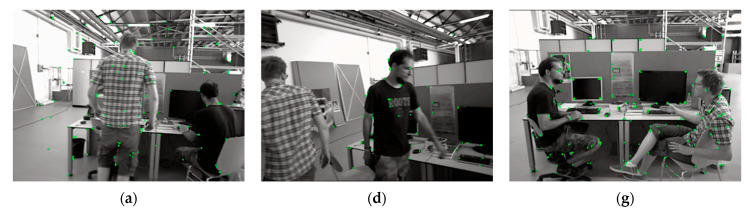

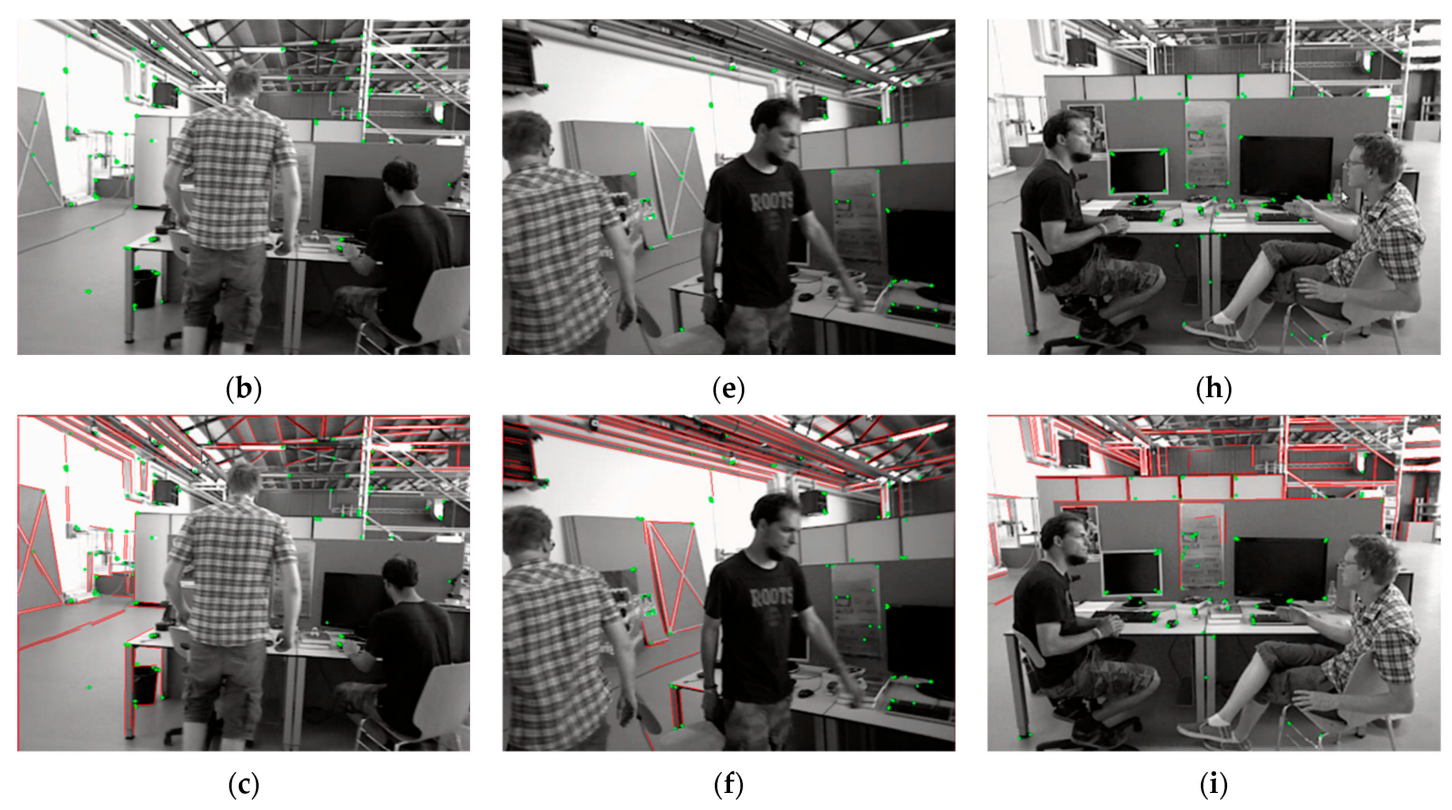
The extraction of feature points, the filtering of dynamic feature points, and the extraction of the non-dynamic region’s line features are compared. (**a**,**d**,**g**) are extracted feature points, where the green dots represent the extracted feature points; (**b**,**e**,**h**) are the effects after removing the dynamic feature points; and (**c**,**f**,**i**) are the extracted non-dynamic region’s line features, where the red lines represent the extracted line features.

We have extracted the ORB feature points in the image and divided the image into dynamic and potential dynamic regions, which will result in some feature points being filtered out. We improve the stability of the algorithm by extracting the line features in the static region again. In this study, we use the LSD algorithm based on the gradient direction and gradient magnitude of the pixel points to extract the line segments. Then, we use the baseline descriptor to represent the line segment feature information, calculate the LBD vector value of each line feature for the adjacent two frames of the image, and use the nearest neighbor method to solve the line feature matching pairs. When the LBD ratio value of the line feature matching pair is greater than a set threshold value, the line feature matching pair will be retained; otherwise, it is rejected as a mismatch. Finally, the weighted fusion strategy based on the ratio of the image’s dynamic region and the number of successful feature point–line matches assigns respective weights to the point–line errors in the cost function to solve the problem of accuracy degradation due to the direct fusion of different types of data errors. Figure 4 shows the results of our algorithm for different processes performed on the same image frame, visually comparing the different effects of the three steps of extracting the feature points in the image, removing the dynamic feature points, and extracting the line features in the non-dynamic region.

We can see that extracting the feature points in the image leads to the extraction of many dynamic feature points, which seriously affects the accuracy of the algorithm. After performing the removal of dynamic points, if the view has a high percentage of dynamic objects, this will filter out some of the feature points, leading to a reduction in the number of extracted points, which reduces the accuracy of the algorithm or causes a tracking failure. By extracting the line features in the non-dynamic region of the image, the problem of missing feature points can be eliminated to better ensure the stability of the algorithm.

### 3.5. Point and Line Fusion

A line segment has four degrees of freedom in the three-dimensional coordinate system, so the combination of the Plücker coordinates and orthogonal representation is used to represent the spatial line segment. We define $P_{1}$ and $P_{2}$ as two points on a straight line in the space, and the spatial straight line Plücker coordinates can be expressed as

(5) $$L_{w}=\left[\begin{array}{c} P_{1}\times P_{2}\\ w_{1}P_{1}-w_{2}P_{2} \end{array}\right]=\left[\begin{array}{c} n\\ v \end{array}\right]$$

where $w_{1}$ and $w_{2}$ are chi-square factors, ν is the direction vector of the line, and n is the normal vector of the plane formed by the coordinate origin and $P_{1}P_{2}$.

The straight line is converted from the world coordinate system to the camera coordinate system and then represented using the Plücker coordinate system.

(6) Lc=TcwLw=Rcw(tcw)^Rcw0R cwnwvw=ncvc

where $L_{c}$ represents a straight line in the camera coordinate system in Plücker coordinates, $L_{w}$ represents a straight line in the world coordinate system in Plücker coordinates, $T_{cw}$ is the transformation matrix from the world coordinate system to the camera coordinate system, $R_{cw}$ is the rotation matrix, $t_{cw}$ is the translation matrix, and tcw^ represents the antisymmetric matrix of $t_{cw}$.

By projecting $L_{c}$ onto the image plane coordinate system, we obtain

(7) $$l_{c}=\left[\begin{array}{ccc} f_{x} & 0 & 0\\ 0 & f_{y} & 0\\ -f_{x}c_{x} & -f_{x}c_{y} & f_{x}f_{y} \end{array}\right]n_{c}=Kn_{c}$$

where $l_{c}$ represents the coordinates of the line in the image plane coordinate system. $f_{x}$, $f_{y}$, $c_{x}$, and $c_{y}$ are the camera’s internal reference, and K is the camera’s internal reference matrix.

The camera position $T_{cw}^{k}$, 3D spatial point $X_{w,i}^{k}$, and line $L_{w,i}^{k}$ are taken as vertices, and their relationships are taken as edges to construct the graph optimization model. It can be determined that the error in the camera position–3D point and the camera position–spatial line segment is

(8) $$\left\{\begin{array}{c} e_{p,i}^{k}=x_{m,i}^{k}-I\left(KT_{cw}^{k}X_{w,i}^{k}\right)\\ e_{l,j}^{k}=d\left(l_{m,j}^{k},KT_{cw}^{k}L_{w,i}^{k}\right) \end{array}\right.$$

where $e_{p,i}^{k}$ represents the projection error of the feature point, $x_{m,i}^{k}$ represents the corresponding pixel coordinates of the feature point, and I is the projection function, which projects the 3D point onto the 2D image plane. $e_{l,j}^{k}$ is the projection error of the line feature, and d is used to compute the line segment error between the measured value and the computed value, which is based on the distance of the endpoints of the line segment as a measure of the error.

Setting the observation error as a Gaussian distribution, we can obtain the following cost function:

(9) $$C=\lambda_{p}\sum_{k,i}\rho_{p}\left(e_{p,i}^{k}{\;\;}^{T}\sum x_{k,i}^{-1}e_{p,i}^{k}\right)+\lambda_{l}\sum_{k,j}\rho_{l}\left(e_{p,j}^{k}{\;\;}^{T}\sum l_{k,j}^{-1}e_{l,j}^{k}\right)$$

where $\lambda_{p}$ and $\lambda_{l}$ are the observation error weighting coefficients of the point and line features, respectively; $\sum x_{k,i}$ and $\sum x_{l,i}$ denote the observation covariances of the point and line features; and $\rho_{p}$, $\rho_{l}$ are the robust kernel functions used to suppress the outliers, which are computed by the following formula:

(10) $$\rho_{m}=\left\{\begin{array}{l} \begin{array}{cc} \frac{1}{2}e_{m,i}^{k}{\;\;}^{2} & \; \end{array}\begin{array}{c} \;\left|e_{m,i}^{k}\right|\leq \delta \end{array}\\ \begin{array}{cc} \delta \left(\left|e_{m,i}^{k}\right|-\frac{1}{2}\delta \right) & other \end{array} \end{array}\right.\begin{array}{cc} , & m\in \{p,l\} \end{array}$$

The weight coefficients $\lambda_{p}$ and $\lambda_{l}$ are set according to Equations (11) and (12). We synthesize and score each image to obtain $M_{p}^{k}$. The integrated score $M_{p}^{k}$ of the image consists of two parts: the ratio of the number of successful feature point–line matches $r_{r}^{k}$ and the image dynamic score $s_{r}^{k}$.

(11) $$\left\{\begin{array}{l} r_{r}^{k}=N_{{\;\;}_{l}}^{k}/N_{p}^{k}\\ M_{p}^{k}=\beta r_{r}^{k}+s_{r}^{k}\\ s_{r}^{k}=\mu S_{d}^{k}/S_{0}\\ \beta =\frac{1}{e^{-N_{p}^{k}+1}} \end{array}\right.$$

where $S_{d}^{k}$ is the area of the dynamic region of the image in the kth frame, $S_{0}$ is the total area of the image, and μ and β are the adaptive scale factors, where β is positively correlated with $N_{p}^{k}$. The current kth keyframe image successfully matches the number of line features $N_{l}^{k}$ and the number of point features $N_{p}^{k}$. $r_{r}^{k}$ is the ratio of line features to point features that are successfully matched.

(12) $$\lambda_{p}=\left\{\begin{array}{cc} \bar{M_{p}^{k}} & \bar{r_{r}^{k}}\leq r\\ 1.25 & \bar{r_{r}^{k}}>r \end{array}\begin{array}{cc} , & \lambda_{l}=\left|1-\lambda_{p}\right| \end{array}\right.$$

where $\bar{M_{p}^{k}}$ is the average of the combined scores of the images, and $\bar{r_{r}^{k}}$ is the average of the ratios of line features and point features extracted from the images. r is a threshold value that is used to control the growth of the coefficients in highly dynamic scenarios.

## 4. Results

In this section, we describe the use of publicly available datasets to validate our algorithm’s effectiveness. Additionally, we compare our SLAM system with state-of-the-art systems from other countries, assessing both their accuracy and real-time performance.

### 4.1. Dataset and Algorithms for Comparison

YPL-SLAM is a SLAM system designed for dynamic scenes, where RGB-D images are passed into the system and used to achieve simultaneous localization and map construction. Therefore, we used the publicly available TUM dynamic object dataset for the system’s evaluation. This dataset was captured by a Kinect camera using the TOF technique and contains multiple color and depth images at a 640 × 480 resolution. The dataset is divided into two classes based on the scene dynamics: class ‘w’ represents walking scenes, which show high dynamic activity, and class ‘s’ represents sedentary scenes, which show lower dynamic activity. The scenes include two people sitting at a table talking and making gestures. The data were further categorized into four modes of camera motion: (1) the static mode, in which the sensor was manually fixed in one position; (2) the xyz mode, in which the sensor was moved along the x, y, and z axes, with the orientation held constant; (3) the hemispherical mode, in which the sensor was moved in a hemispherical space with a diameter of approximately one meter; and (4) the rpy mode, in which the sensor was in the same position around a major axis’ (roll, pitch, yaw) rotation.

The YPL-SLAM algorithm uses these datasets for comparison with other algorithms. When comparing the algorithms, we consider that YPL-SLAM is derived from the improvements in ORB-SLAM2, which excels in static environments. Hence, we select ORB-SLAM2 for comparison. In dynamic scenes, where Dyna-SLAM and DS-SLAM are recognized as advanced open-source SLAM systems, we include them in the comparison to demonstrate the performance of our proposed algorithm.

### 4.2. Evaluation Method

We utilize the open-source assessment tool evo to compare the performance of the algorithms. Evo offers a range of utilities for the assessment of SLAM system scripts and measurements, enabling the comparison of different algorithms. It supports evaluations using real-world datasets and provides visualization tools for the analysis of SLAM systems’ outputs. The absolute trajectory error (ATE) serves as a metric to evaluate SLAM systems’ performance. It quantifies the difference between the trajectories output by the SLAM algorithm and the true or reference trajectories, aiding in the assessment of the system’s robustness and accuracy across varying environments and over extended periods. Additionally, we compare the relative pose errors of the different algorithms, which are commonly used to evaluate the drift in displacement estimation.

The following equation is primarily used in calculating the root mean square error of the absolute trajectory error (ATE) between the estimated pose E and the ground truth pose G.

(13) $$ATE-RMSE=\sqrt{\frac{1}{n}\sum_{i=0}^{n}||trans(E)-trans(G)|{|}_{2}^{2}}$$

In this equation, n represents the total number of poses to be computed, $trans\left(G\right)$ represents the true pose of the camera, $trans\left(E\right)$ represents the estimated pose of the camera, and ||·|| denotes the Euclidean distance between these two poses.

The formula for *RPE-RMSE* is as follows:

(14) $$RPE-RMSE=\sqrt{\frac{1}{m}\sum_{i=1}^{m}\left||trans{\left(Q_{i}^{-1}Q_{i+\Delta }\right)}^{-1}(P_{i}^{-1}P_{i+\Delta }\right)|{|}_{2}^{2}}\begin{array}{cc} , & m=n-\Delta \end{array}$$

where $Q_{i}$ denotes the ground truth pose, $P_{i}$ denotes the estimated pose, and Δ denotes the time interval between two consecutive poses.

We evaluate the performance of our algorithm, YPL-SLAM, compared to ORB-SLAM2, using the following equation:

(15) $$\eta =\frac{\sigma -\tau }{\sigma }\times 100\%$$

The above equation represents the performance comparison between our algorithm and ORB-SLAM2, where σ denotes the error generated by ORB-SLAM2 and τ denotes the error generated by the YPL-SLAM algorithm.

### 4.3. Tracking Accuracy Evaluation

**Figure 5:**
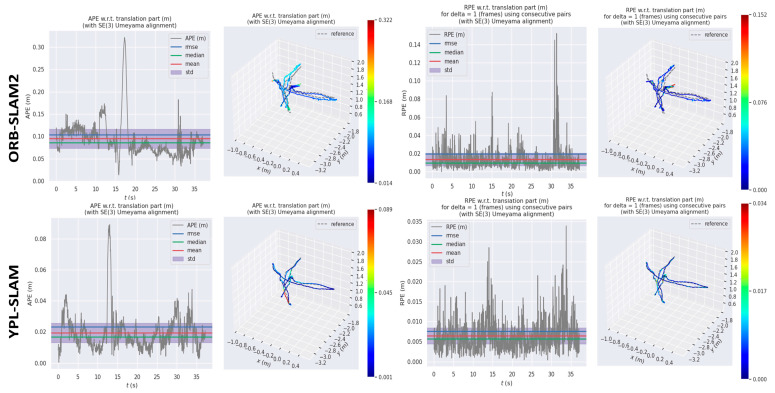
In the fr3_s_hs sequence, we compare the absolute trajectory error (ATE) and relative pose error (RPE) between ORB-SLAM2 and YPL-SLAM, particularly focusing on the translation drift. The first and second columns of the table display the ATE results, while the third and fourth columns show the RPE results.

**Figure 6:**
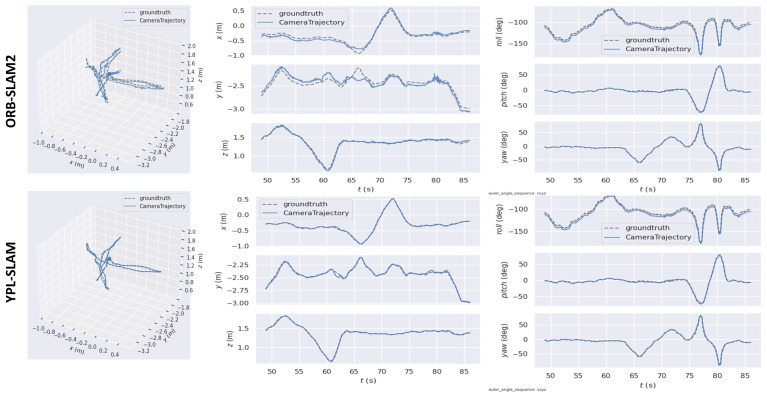
In the fr3_s_hs sequence, we compare the ground truth and estimated trajectories. The first column displays the comparison of the 3D trajectories, containing the ground truth and trajectories estimated by both ORB-SLAM2 and YPL-SLAM. The second column presents the fitting results for the X, Y, and Z axes, while the third column shows the fitting results for the roll, pitch, and yaw axes.

Figure 5 illustrates the absolute trajectory error (ATE) and relative pose error (RPE) values of ORB_SLAM2 and YPL-SLAM, computed using the evo tool in low dynamic scenes. Notably, YPL-SLAM demonstrates a lower ATE and RPE, indicating higher accuracy compared to ORB_SLAM2 in such scenarios. Furthermore, Figure 6 provides a comparison of the trajectories estimated by YPL-SLAM with the real trajectories. It shows that the trajectories estimated by YPL-SLAM closely align with the ground truth trajectory. An examination of the X, Y, and Z axes, as well as the pitch, yaw, and roll views, reveals that the estimated results of YPL-SLAM closely match the ground truth data.

**Figure 7:**
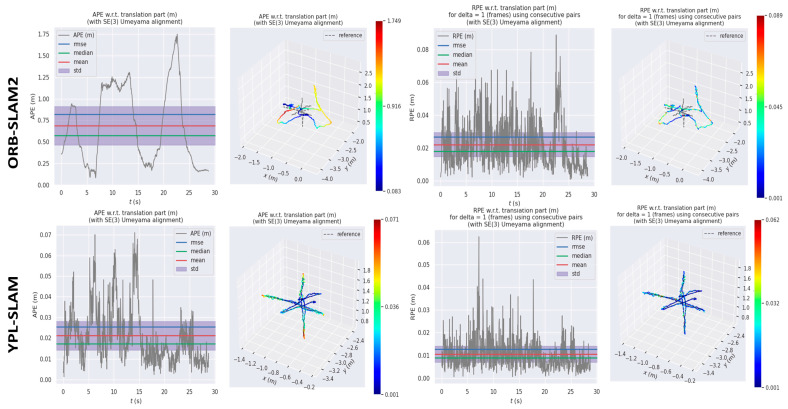
In the fr3_w_xyz sequence, we compare the absolute trajectory error (ATE) and relative pose error (RPE) between ORB-SLAM2 and YPL-SLAM, focusing on the translation drift performance. The first two columns of the table display the ATE results, while the third and fourth columns show the RPE results.

**Figure 8:**
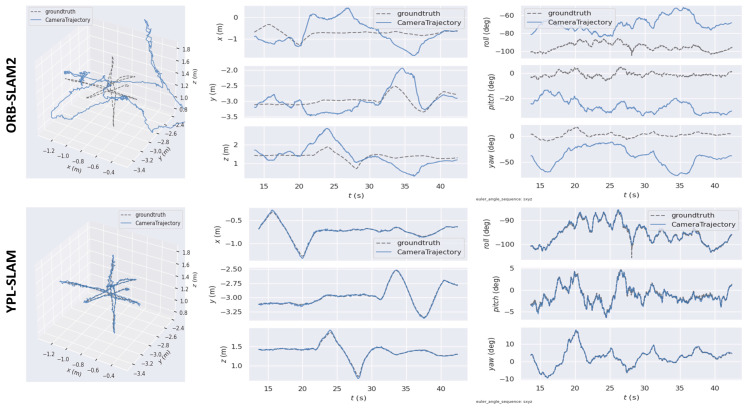
In the fr3_w_xyz sequence, we compare the ground truth and estimated trajectories. The first column displays the comparison of the 3D trajectories, containing the ground truth and trajectories estimated by both ORB-SLAM2 and YPL-SLAM. The second column presents the fitting results for the X, Y, and Z axes, while the third column shows the fitting results for the roll, pitch, and yaw axes.

Figure 7 and Figure 8 depict images captured in highly dynamic scenes, showcasing the stability and effectiveness of YPL-SLAM in such environments. These images offer a detailed visual representation of YPL-SLAM’s performance in the context of highly dynamic environments. Overall, these results demonstrate that YPL-SLAM achieves satisfactory localization performance in both less and highly dynamic scenes.

Table 2, Table 3 and Table 4 present the quantitative results of the comparison of YPL-SLAM with other algorithms in terms of ATE and RPE. The root mean square error (RMSE) and standard deviation (SD) are reported in the tables to better illustrate the localization accuracy and system stability. The RMSE and SD provide a more intuitive understanding of the accuracy of the results. Table 2 illustrates that the ATE calculated for YPL-SLAM improves across all sequences. Compared to ORB-SLAM2, YPL-SLAM shows notably enhanced performance in highly dynamic scenes, achieving a significant improvement in accuracy by an order of magnitude. Particularly in the fr3_w_xyz sequence, which represents a highly dynamic scenario, the average error and root mean square error show the most substantial improvement, reaching 96.1% and 95.76%, respectively. This indicates that YPL-SLAM effectively enhances the system’s performance in highly dynamic environments, particularly in terms of accuracy and stability. In less dynamic environments, YPL-SLAM also exhibits significant improvements over ORB-SLAM2, yielding very favorable results. However, when compared to Dyna-SLAM and DS-SLAM, the difference in the performance of YPL-SLAM is not significant.

Table 3 and Table 4 present the results of the relative pose error (RPE) comparison between YPL-SLAM and the other algorithms. Table 3 displays the results for the translational part of the RPE, while Table 4 shows the results for the rotational part of the RPE. Compared with ORB-SLAM2, YPL-SLAM shows an improvement in the RMSE and SD in the translational part of the RPE of 74.75% to 88.63% and 77.45% to 91.56%, respectively, and in the rotational part of the RPE by 70.66% to 86.26% and 74.97% to 89.38, respectively, in highly dynamic sequences. The experimental results for YPL-SLAM are superior in some sequences compared to those of Dyna-SLAM and DS-SLAM. Through numerous tests, we have observed that YPL-SLAM achieves stability and reliability in highly dynamic sequences.

### 4.4. Real-Time Evaluation

To assess the real-time performance of YPL-SLAM, a series of comparative experiments involving ORB-SLAM2, Dyna-SLAM, and YPL-SLAM were conducted, processing each image frame fully. YPL-SLAM and Dyna-SLAM were tested on identical computers equipped with an Intel i7-12700 CPU and an NVIDIA RTX 2060 graphics card to ensure a fair comparison. To evaluate the real-time performance on the same hardware, we compared the processing speeds of Dyna-SLAM and YPL-SLAM. YPL-SLAM utilizes YOLOv5s for target detection and employs parallel processing, making its processing time largely independent of the target detection time. However, in the tracking thread, it is necessary to utilize the results of target detection to extract line features in static regions. Therefore, we add the processing time for target detection to the processing time of each frame. Table 5 presents the experimental results. Despite increasing the overhead for target detection, YPL-SLAM maintains good real-time performance compared to ORB-SLAM2. Furthermore, compared to Dyna-SLAM, YPL-SLAM demonstrates stronger real-time performance. This indicates that YPL-SLAM can operate more efficiently when combining target detection and line feature extraction, providing an advantage in quickly recognizing and removing dynamic objects in real-time applications.

## 5. Discussion

In this study, we proposed a SLAM method to cope with point–line feature functions in dynamic environments: YPL-SLAM. Compared with ORB-SLAM2, YPL-SLAM adds target identification and region delineation threads, as well as a line feature extraction module and a point–line fusion optimization module. Through its comparison with other SLAM algorithms in the dynamic sequences of the TUM dataset, we obtained the following insights:

- Compared with the ORB-SLAM2 method, the performance of YPL-SLAM in a highly dynamic environment is more obvious, and the localization accuracy is improved by more than 80%.
- In comparison with Dyna-SLAM and DS-SLAM, YPL-SLAM still has some shortcomings in highly dynamic scenarios, although the performance difference is not significant in most cases. These shortcomings are mainly due to the fact that Dyna-SLAM utilizes the a priori dynamic region information of each frame for pixel-level semantic segmentation, which results in superior dynamic point filtering capabilities.
- Comparing the RPE results, it can be seen that the computation results obtained via point–line fusion have fewer errors. The point–line fusion approach plays an optimizing role in the calculation of the relative pose and reduces the error level of the system. It has a significant effect in terms of improving the accuracy and reliability of the attitude estimation process.
- Based on its ability to guarantee high accuracy, the speed of YPL-SLAM ensures its real-time operation. Moreover, by utilizing YOLOv5s for target recognition and the division of the region where the target is located, and by extracting the line features of the static region, the robustness and accuracy of the system are guaranteed.

## 6. Conclusions

Addressing the situation in which removing dynamic feature points in dynamic environments with multiple moving objects may lead to a reduction in the number of feature points, which in turn leads to the loss of the object, a new SLAM method, YPL-SLAM, is proposed. It is based on ORB-SLAM2 and utilizes YOLOv5s to perform target recognition. This method divides the target’s area to determine the dynamic region, and the dynamic region’s feature points are removed; then, it extracts the line features of the non-dynamic region and uses a weighted fusion strategy considering the image dynamic scores and the number of successful feature point–line matches for point–line fusion optimization and processing, takes advantage of the high robustness of the line features in the texture-deficient or motion-blurring scenarios, ensuring the stability and accuracy of the system. Compared with the ORB-SLAM2 method, the performance improvement provided by YPL-SLAM in highly dynamic environments is more obvious, and the absolute trajectory error is reduced by up to 96.1% in the highly dynamic sequences of the TUM dataset. In the optimization process of point–line fusion, a weighted fusion strategy that combines the dynamic score of the image and the number of successful feature point–line matches is used for the point–line fusion optimization process. This solves the problems of reducing the number of feature points, resolving the issue of lost objects to a certain extent, and improving accuracy. Meanwhile, the speed of YPL-SLAM can ensure real-time operation.

Although YPL-SLAM has allowed significant progress in processing dynamic scenes, we believe that there is potential for further improvement. In our study, the scene tests in the TUM dataset were conducted under good lighting conditions. Given the critical role of illumination in vision SLAM, we plan to integrate vision and LiDAR in our subsequent work to fully utilize the advantages of LiDAR in poor lighting conditions. The point cloud data provided by LiDAR are insensitive to light, which will help to compensate for the shortcomings of vision SLAM in light-complex scenes. By combining the information from vision and LiDAR, we expect to improve the stability of the SLAM algorithms under various environmental conditions, which will bring greater performance improvements for real-time localization and map building. At the same time, we will consider introducing strategies that enable the system to adapt to different environmental conditions to ensure good performance in various scenarios. Mechanisms for the automatic adjustment of the algorithm’s parameters could be developed so that real-time adjustments can be made at runtime in response to changes in the environment. Such a strategy will enable the system to adapt to changes in illumination, scene complexity, and dynamics, thus improving its robustness. This integrated approach is expected to further advance SLAM’s performance in dynamic scenes and under complex lighting conditions.

## Figures and Tables

**Table 1:** Regional division by target category.

Region Name	Target-Semantic Information
Potential dynamic region	keyboards, chairs, books, mouses, etc.
Dynamic region	people, animals, mobile robots, etc.
Static region	computer screens, cabinets, windows, etc.

**Table 2:** Experimental results for absolute trajectory error (ATE [m]).

Sequences	ORB-SLAM2		Dyna-SLAM		DS-SLAM		YPL-SLAM		Improvement against ORB-SLAM2	
	RMSE	SD	RMSE	SD	RMSE	SD	RMSE	SD	RMSE (%)	SD (%)
**fr3_s_hs**	0.066009	0.035507	0.028754	0.014252	0.027626	0.013821	0.028696	0.013893	56.53	60.87
**fr3_w_hs**	0.508235	0.234165	0.027093	0.01329	0.03139	0.016229	0.027681	0.013727	94.55	94.14
**fr3_w_rpy**	0.760449	0.37388	0.044161	0.021385	0.348257	0.235303	0.044471	0.025282	94.15	93.24
**fr3_w_static**	0.059643	0.034103	0.010198	0.004706	0.008354	0.003663	0.009897	0.004225	83.41	87.61
**fr3_w_xyz**	0.681858	0.339961	0.032547	0.017512	0.026653	0.016354	0.026567	0.014415	96.1	95.76

**Table 3:** Experimental results for translational relative trajectory error.

Sequences	ORB-SLAM2		Dyna-SLAM		DS-SLAM		YPL-SLAM		Improvement against ORB-SLAM2	
	RMSE	SD	RMSE	SD	RMSE	SD	RMSE	SD	RMSE (%)	SD (%)
**fr3_s_hs**	0.035759	0.023872	0.024337	0.013665	0.023779	0.013261	0.016538	0.009941	53.75	58.36
**fr3_w_hs**	0.16326	0.130115	0.023624	0.012429	0.029535	0.015364	0.023474	0.012247	85.62	90.59
**fr3_w_rpy**	0.17649	0.135735	0.035798	0.020339	0.144131	0.106249	0.044562	0.030602	74.75	77.45
**fr3_w_static**	0.047703	0.041116	0.009643	0.004707	0.010264	0.005483	0.009058	0.004898	81.01	88.09
**fr3_w_xyz**	0.170089	0.115373	0.020675	0.01056	0.019706	0.011965	0.019335	0.009732	88.63	91.56

**Table 4:** Experimental results for rotational relative trajectory error.

Sequences	ORB-SLAM2		Dyna-SLAM		DS-SLAM		YPL-SLAM		Improvement against ORB-SLAM2	
	RMSE	SD	RMSE	SD	RMSE	SD	RMSE	SD	RMSE (%)	SD (%)
**fr3_s_hs**	0.016118	0.008231	0.015482	0.0074	0.016714	0.008962	0.013464	0.006323	16.47	23.18
**fr3_w_hs**	0.084237	0.064951	0.015727	0.008289	0.019449	0.009518	0.015651	0.008309	81.42	87.21
**fr3_w_rpy**	0.086723	0.065983	0.018861	0.010664	0.065916	0.036591	0.02466	0.016516	71.56	74.97
**fr3_w_static**	0.02128	0.017428	0.006506	0.002736	0.006635	0.002994	0.006244	0.002915	70.66	83.27
**fr3_w_xyz**	0.081139	0.053901	0.011265	0.005714	0.010372	0.005463	0.011149	0.005722	86.26	89.38

**Table 5:** Experimental results for tracking time(s).

Sequences	ORB-SLAM2	Dyna-SLAM	YPL-SLAM	Improvement against Dyna-SLAM (%)
**fr3_s_hs**	0.0191	1.7173	0.0791	95.39%
**fr3_w_hs**	0.0204	1.8274	0.0732	95.99%
**fr3_w_rpy**	0.0201	1.7785	0.0772	95.66%
**fr3_w_static**	0.0183	1.7647	0.0735	95.83%
**fr3_w_xyz**	0.021	1.7642	0.078	95.58%

## Data Availability

The research data used in this paper are from https://vision.in.tum.de/data/datasets/rgbd-dataset/download (accessed on 11 April 2023).
